# Factors Deciding the Sex of the Offspring

**Published:** 1884-04-20

**Authors:** 


### FACTORS DECIDING THE SEX OF THE OFFSPRING.

Many a theory has been proposed—and exploded—concerning the conditions which decide the sex in the offspring. Those interested in the raising of horses, cattle, pigs, etc., have doubtless made a more practical progress in this question than physicians have done with regard to the human being. It is not rare to notice that a sheep-grower, for instance, will raise only ewes, if it is his intention to do so.

Recently, Dr. K. Dusing has studied the factors deciding the sex of the fœtus, and published (Centrlbl. f. d. Med. Wiss. 45, 1883,) an interesting article concerning his results; and as the subject has an undoubted vital bearing upon the propagation of the human race, we will not withhold it from our readers.

Fiquet made first the observation on cattle, that those animals that sexually were very much occupied produced more individuals of their own sex, and D. explains this by saying that the male whose genital apparatus is frequently used is provided with fresher sperma, so that fecundation ensues with comparatively young and active spermatozoa. In such a case more male offspring are produced; while a male whose sexual organs rarely perform their function will give rise to more female offspring because his spermatozoa are older, weaker. In the female the same holds good, the ova being earlier (fresher) fecundated, and a predisposition to the female sex takes place. As a natural consequence, it follows that the more there is a want of individuals of one sex, the more their sexual functions are required—the quicker, the younger their sexual product is made use of—the more members of that sex will be born. It is simply the law of nature to propagate the species.

The same effect as sexual over-exertion is also produced by deficient nutrition, if the same demands are made upon the genital apparatus. Weak bulls paired with vigorous cows produced invariably male calves, while weak cows paired with vigorous bulls gave mainly birth to female calves (Tellari and Fiquet).

Next of importance is the age. At the time of the greatest sexual power, the individual of each sex never transfers his (or her) sex to that of his (or her) offspring, or does at least much less so. In most human families there are, then, found the most boys if the husband is much older than the wife. (Hofacker, Sadler.) It shows the intention of nature to produce more individuals of that sex which threatens to die out; the weaker, the older the propagator, the more care nature takes to have his sex propagated.

Thus far the influences concerned one sex. In case the disturbances of nutrition affect both parties the rule holds good that where there is a superfluity of nutrition the reproduction is stronger; where a want, it is weaker. The genital system is first influenced by nutrition. (Darwin.) But it is especially the female who is more dependent upon nutrition, probably because she has to care for the bringing-up of the offspring. The logical conclusion is, that in case of superfluity of nutrition more females are born; in case of want, more males. Thus Ploss found that in the same ratio as the price of provisions advanced, the number of male children increased. Or may not the cause be the following? It is the male that has to work hard; it is the male that suffers soonest by bad nutrition; the male, therefore, becomes soonest the weaker; therefore, the result.

Laudvis procured from thousands of very young caterpillars of vanessa urticæ males or females, whatever he preferred, by nourishing them either badly or well. In some of the lower animals, the females find their nourishment mainly in summer, the males in winter. In these more females are born in summer, the males in winter.

D. draws from these observations the conclusions: The sexual relation regulates itself by the property of animals and plants to produce that sex in greater numbers whose relatively greater augmentation is profitable for the propagation of the species. Even an anomalous sexual relation may benefit under special circumstances the propagation. The sexual difference may rest in the unimpregnated ovum (for instance, tendency of young ova to female sex), or the sex is determined at fecundation (young spermatozoa, males), or the nutrition (strength, age, food) decides.

Although these theories find an undeniable proof in observations and experiments made on animals (and some on human beings, also), we have to be very cautious in admitting their truth. Other factors, at present unknown, probably determine, besides those mentioned, the sex of the fœtus. It is even doubtful whether the latter is already definitely decided at fecundation, and whether later influences may not have their share in ordaining the sex.—*Med. and Surg. Rep.*

